# Development of β Type Ti23Mo-45S5 Bioglass Nanocomposites for Dental Applications

**DOI:** 10.3390/ma8125441

**Published:** 2015-11-25

**Authors:** Karolina Jurczyk, Andrzej Miklaszewski, Mieczyslawa U. Jurczyk, Mieczyslaw Jurczyk

**Affiliations:** 1 Department of Conservative Dentistry and Periodontology, Poznan University of Medical Sciences, Bukowska 70, Poznan 60-812, Poland; karolajur@gmail.com; 2 Institute of Materials Science and Engineering, Poznan University of Technology, Jana Pawla II 24, Poznan 61-138, Poland; andrzej.miklaszewski@put.poznan.pl; 3 Division of Mother’s and Child’s Health, Poznan University of Medical Sciences, Polna 33, Poznan 60-535, Poland; mujurczyk@gmail.com

**Keywords:** titanium, 45S5 bioglass, nanocomposite, powder metallurgy, surface properties, etching

## Abstract

Titanium β-type alloys attract attention as biomaterials for dental applications. The aim of this work was the synthesis of nanostructured β type Ti23Mo-*x* wt % 45S5 Bioglass (*x* = 0, 3 and 10) composites by mechanical alloying and powder metallurgy methods and their characterization. The crystallization of the amorphous material upon annealing led to the formation of a nanostructured β type Ti23Mo alloy with a grain size of approximately 40 nm. With the increase of the 45S5 Bioglass contents in Ti23Mo, nanocomposite increase of the α-phase is noticeable. The electrochemical treatment in phosphoric acid electrolyte resulted in a porous surface, followed by bioactive ceramic Ca-P deposition. Corrosion resistance potentiodynamic testing in Ringer solution at 37 °C showed a positive effect of porosity and Ca-P deposition on nanostructured Ti23Mo 3 wt % 45S5 Bioglass nanocomposite. The contact angles of glycerol on the nanostructured Ti23Mo alloy were determined and show visible decrease for bulk Ti23Mo 3 wt % 45S5 Bioglass and etched Ti23Mo 3 wt % 45S5 Bioglass nanocomposites. *In vitro* tests culture of normal human osteoblast cells showed very good cell proliferation, colonization, and multilayering. The present study demonstrated that porous Ti23Mo 3 wt % 45S5 Bioglass nanocomposite is a promising biomaterial for bone tissue engineering.

## 1. Introduction

Since 1965, titanium has found an increasing application as an implant material in medicine. Commercial purity titanium has high corrosion resistance and outstanding biocompatibility [1,2,3]. One reason for these advantages may be a protective oxide layer, which forms spontaneously on the implant surface [4]. Titanium exists in two allotropic forms. At low temperatures, it has a closed packed hexagonal crystal structure (hcp), which is commonly known as α, whereas above 882 ± 2 °C, it has a body centered cubic structure (bcc) termed β. The alloying elements such as Al, O, N, *etc.* tend to stabilize the α phase while elements V, Mo, Nb, Fe, Cr, *etc.* stabilize the β phase [1,3].

Titanium and its alloys also attract a lot of attention in dental applications [5,6,7]. Pure titanium and Ti-6Al-4V alloy are the main materials in the dental field as well as in the surgical one. Implant sensitivity to titanium alloys is very seldom, despite components such as vanadium which are described to be cytotoxic [7]. By the elimination of toxic elements, it is possible to prepare Ti-type alloys with excellent biocompatibility. Ti-6Al-7Nb, which has been developed for surgical implants, is also attractive for dental applications [8]. Recently, Ti-40Zr, Ti-5Al-13Ta and Ti-43.1Zr-10.2Al-3.6V have been proposed [9,10]. Moreover the development of Ti-Mo-A (A = Ga, Ge, Al) and Ti-Ta, Ti-Ta-Zr, Ti-Sc-Mo alloys, as shape memory titanium alloys, for biomedical use is noticeable [11]. All above mentioned are β type titanium alloys.

New perspectives appear with nanostructure materials, which exhibit better mechanical and physico-chemical properties in comparison to their microcrystalline counterparts [12,13,14]. Current goals in the development of new Ti-based biomaterials are: (i) to avoid potentially toxic elements, such as vanadium, to further improve biocompatibility; (ii) to produce titanium alloys with a high fatigue strength. β-titanium alloys partially fulfill these requirements [1,5,8,9,10,11]. One of these improved β titanium alloys is Ti12Mo6Zr2Fe, which is currently used for implant materials.

Dental implants for load bearing applications require the use of materials that are both bioactive and have significant mechanical strength [8]. There are no existing materials that readily fit these two criteria. While titanium and titanium-based alloys have excellent mechanical properties and are generally well tolerated in a physiological environment, they have negligible capacity for osteointegration. Two approaches have been made to improve the osteointegration: a surface modification approach and a composite approach [15,16]. In the surface modification approach, the surface of the Ti surgical substrates is modified by chemical or electrochemical methods to make the surface bioactive, or a bioactive coating is applied using, for example, electrophoretic deposition technique (EPD) [15]. In the composite approach, the primary material is combined with bioactive materials, such as 45S5 Bioglass. Conventional powder metallurgy methods [14,16] or plasma-assisted processes [17] are commonly used for fabricating composites.

The biocomposites prepared from powder mixtures of titanium (α-Ti), hydroxyapatite (HA), and bioactive glass (BG) (SiO_2_-CaO-P_2_O_5_-B_2_O_3_-MgO-TiO_2_-CaF_2_) were investigated [18]. The results showed that complex reactions among the starting materials mainly depended on the initial Ti/HA ratios as well as the sintering temperatures.

Recent studies in nanometer sized bioactive glasses (nBG) have indicated their possibility for biomedical applications. The potential of top-down processing of 45S5 BG by wet comminution in a stirred media mill was investigated [19]. The products were assessed by *in vitro* hydroxy-carbonate apatite (HCAp) formation in simulated body fluid, which is a marker for bioactive behavior. Bioactive submicron particles can be produced in organic solvents (*n*-butanol, *n*-pentanol, *n*-hexanol). However bioactivity is lost after processing in water. Wet comminution under controlled conditions is an appropriate technique in the production of submicrometer bioactive glass particles with enhanced bioactivity.

Moreover, bioceramic (HA, 45S5 Bioglass) coatings, applied in order to improve the surface bioactivity of titanium and its alloys, often flake off as a result of poor ceramic/metal interface bonding, which may lead to failure of the surgery [20]. The problems mentioned above can be solved by the fabrication of Ti-bioceramic composites. Recently the studies on the fabrication and characterization of Ti-based composites reinforced with bioceramic (HA or 45S5 Bioglass) particles have been reported [16,21,22].

The properties of composites depend very much upon structure, shape, volume fraction, and the interface among the constituents, as well as upon the chemical composition and their final microstructure. The *in vivo* studies indicate that Ti-20 vol % HA composite has good biocompatibility and can integrate with bone [22]. The osteointegration ability of the composite is better than that of pure titanium, especially in the early stage after the implantation, which may be due to the presence of HA or 45S5 Bioglass in the Ti-matrix composite [23].

In this work the mechanical alloying (MA) process followed by the pressing and sintering was applied for the preparation of the nanocrystalline β-type Ti23Mo alloy and Ti23Mo-*x* wt % 45S5 Bioglass nanocomposites (*x* = 3 and 10). Finally, the bulk samples were anodically etched. The bioactive ceramic Ca-P layer was cathodically deposited too. Structure, microstructure, composition, porosity, surface morphology, corrosion resistance, wettability, and *in vitro* cytocompatibility were investigated. Yet to the authors’ knowledge, there have been no papers regarding the addition of 45S5 Bioglass into β Ti23Mo nanocomposite to have appeared until now.

## 2. Experimental Details

### 2.1. Chemicals and Materials

The following commercial powders were used: titanium, CAS number 7440-32-6 (<45 μm, 99.9%, Alfa Aesar GmbH & Co KG, Karlsruhe, Germany), molybdenum, CAS number 7439-98-7 (44 μm, 99.6%, Sigma Aldrich, Saint Louis, MO, USA), 45S5 Bioglass (45% SiO_2_, 24.5% Na_2_O, 24.5% CaO, 6% P_2_O_5_, 53 μm; all in wt % from Mo-Sci Health Care L.L.C., Rolla, MO, USA).

The experiments were carried out on Ti23Mo alloy and two composite materials. For brevity, in this work, materials are denoted as follows:

- Ti23Mo nanocrystalline alloy is labeled as Ti23Mo.
- Ti23Mo-3 wt % 45S5 Bioglass nanocomposite is labeled as Ti23Mo 3BG.
- Ti23Mo-10 wt % 45S5 Bioglass nanocomposite is labeled as Ti23Mo 10BG.

### 2.2. Sample Preparation

**Figure 1:**
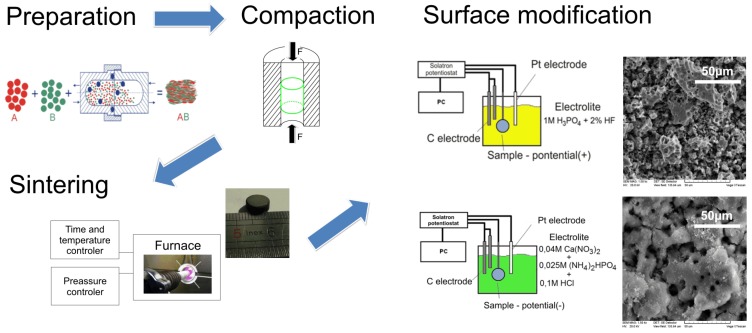
Experimental setup Ti23Mo-*x* wt % 45S5 Bioglass nanocomposite synthesis and electrochemical treatment procedure.

Mechanical alloying (MA) was performed using SPEX 8000 Mixer Mill (SPEX^®^ SamplePrep, Metuchen, NJ, USA). Argon was a protective atmosphere. Round bottom stainless vials were used. Elemental powders (titanium, CAS number 7440-32-6 (<45 μm, 99.9%, Alfa Aesar), molybdenum, CAS number 7439-98-7 (44 μm, 99.6%, Sigma Aldrich) and 45S5 Bioglass (45% SiO_2_, 24.5% Na_2_O, 24.5% CaO, 6% P_2_O_5_, 53 μm; all in wt % from Mo-Sci Health Care L.L.C.)) were weighted, blended and poured into vials in a glove box (Labmaster 130) filled with automatically controlled argon atmosphere (O_2_ < 2 ppm and H_2_O < 1 ppm). The weight ratio of hard steel balls (10 mm diameter) to powder weight ratio equaled 15:1. The MA process lasted 30 h in all cases. In order to prevent severe cold welding during high-energy milling, the ball milling was stopped every 2 h to dissipate the heat and to reduce an excessive rise in temperature. In the next step, the produced powders with size distribution of the particles from 40–150 μm were placed into the matrix and uniaxially pressed at a pressure of 500 MPa. Finally, the green compacts were heated over 1 h to 800 °C and kept at this temperature for 30 min for particle sintering. After that, the sinters were slowly cooled down to room temperature (RT) together with the furnace. The sintering was done at 10^−2^ Pa vacuum in an alumina tube (McDanel Adv. Ceramic Technologies, Beaver Falls, PA, USA). The final bulk sinters have diameter and height of 8 and 4 mm, respectively (Figure 1).

The Solartron 1285 potentiostat (Solatron analytical, Farnborough, UK) was applied in the electrochemical etching stage of the sinters, followed at 10 V *vs.* open-circuit-potential (OCP) for 60 min in 1 M H_3_PO_4_ + 2% HF electrolyte. In the following stage, calcium phosphate was deposited on the surface. We applied cathodic deposition at −5 V *vs.* OCP in the electrolyte containing 0.042 M Ca(NO_3_)_2_ + 0.025 M (NH_4_)_2_HPO_4_ + 0.1 M HCl for 1 h.

### 2.3. Material Characterization

The crystallographic structure of the samples during different processing stages was investigated at room temperature using a Panalytical Empyrean X-ray diffraction (XRD) with CuKα_1_ (λ = 1.54056 Å) radiation (Panalytical, Empyrean model, Almelo, The Netherlands). The conditions of XRD measurements were: voltage 45 kV, anode current 40 mA, 2θ range 20°–80°, time per step 12.54 s/step, step size 0.0167°. The average crystallite sizes d were estimated by Scherrer method: β = 0.9 λ/d cosθ, where β is the full-width at half maximum intensity of a Bragg reflection excluding instrumental broadening, θ the Bragg angle, and λ the wavelength of the X-ray radiation. Scanning electron microscope (SEM, VEGA 5135 Tescan, Brno, Czech Republic) with energy dispersive spectrometer (EDS, PTG Prison Avalon, Princeton Gamma Tech., Princeton, NY, USA) was used to characterize the chemical composition of the prepared samples. EDS was calibrated using a typical Cu calibration procedure. Additionally, the Ti (99.9% from Alfa Aesar), Mo (99.6% from Sigma Aldrich) and SiO_2_, Na_2_O, CaO, P_2_O_5_ (all from Sigma Aldrich) specimens were used as reference.

The porosity of the porous materials was calculated by the formula *p* = (1 − ρ/ρ_th_) × 100%, where ρ and ρ_th_ are the density of the porous material and its corresponding theoretical density calculated for the rule of the mixtures, respectively. The density of the sintered samples was determined by Archimedes method. The Vickers microhardness of the bulk samples was measured using a microhardness tester by applying a load of 300 g for 10 s on the polished surfaces of the samples. For each sample, 10 separate indents were created on the investigated surface.

The surface morphology of the samples was investigated using an Optical Profiler NT 1100 (Veeco WykoR^®^, Mannheim, Germany). Using the 3D optical profiler, the following standard roughness parameters were estimated: arithmetic mean roughness (μm)—*R*_a_, maximum height of the profile (μm)—*R*_t_, ten point mean roughness (μm)—*R*_z_ and hybrid 3D parameters: root mean square (°)—*S*_dq_, surface area ratio gradient (%)—*S*_dr_.

The Solartron 1285 potentiostat (Solatron analytical) was applied for corrosion measurements. The corrosion resistance of different samples was measured in Ringer’s solution (simulated body fluid with composition: NaCl: 9 g/L, KCl: 0.42 g/L, CaCl_2_: 0.48 g/L, NaHCO_3_: 0.2 g/L) applying potentiodynamic mode with scan rate 0.5 mV/s at temperature of 37 ± 1 °C, controlled by thermostat. The corrosion test was run in EG & G K0047 corrosion cell. The counter electrode consisted of two graphite rods, and a platinum electrode (SCE, Hydromet, Gliwice, Poland) was used as the reference electrode. The surface area exposed to the electrolyte was 0.5 mm^2^. Polarization curves were obtained for each specimen in the potential range for −2 to 3 V. The corrosion potentials (*E*_corr_) and corrosion current densities (*I*_corr_) were estimated from the Tafel extrapolations of the corrosion curves, using CorrView software (Scribner Associates Inc., Southern Pines, NC, USA).

For the surface wettability measurements, contact angles Θ were determined by computer software analysis Musial (Elektronika Jadrowa, Krakow, Poland) from geometrical shape of droplets recorded by the optical system with a digital camera. Droplets placement were realized by a special micropipette with a constant volume of test liquid (glycerol (99.5% purity); 2 μdm^3^) on a clear, polished and rinsed with alcohol surface, by it raise until the bottom touches the specimen. If the drop is large enough, the adhesion to the surface pulls it off from the tip. All the data were obtained under equilibrium conditions, procedure was repeated three times for all samples. For each recorded picture (*i.e.*, for each liquid drop), the geometrical shape analysis was repeated 10 times: the extreme values were rejected, and the arithmetic mean value was calculated for the accepted findings.

### 2.4. In vitro Cytocompatibility

The *in vitro* cytocompatibility tests were performed under static conditions. The discs of Ti23Mo 3BG nanocomposite after electrochemical etching and additional Ca-P deposition were sterilized by autoclaving at 120 °C for 15 min and were separately located at the bottom of 24-well microplates. Normal Human Osteoblast (NHOst) cells from Cambrex (CC-2538, Walkersville, MD, USA) were cultured onto each disc at a concentration of 5000 cells/well in 1 mL of culture medium under static conditions. The cells were cultured at 37 °C in a 5% CO_2_ incubator for 1 and 5 days. The medium was replaced every day. Then, the cells were fixed with a 25% glutaraldehyde solution for 10 min and stained with a 10% Giemsa's staining solution for 10 min. The specimens were sputter-coated with gold and examined using scanning electron microscope (SEM).

## 3. Results

### 3.1. Structure Properties

**Figure 2:**
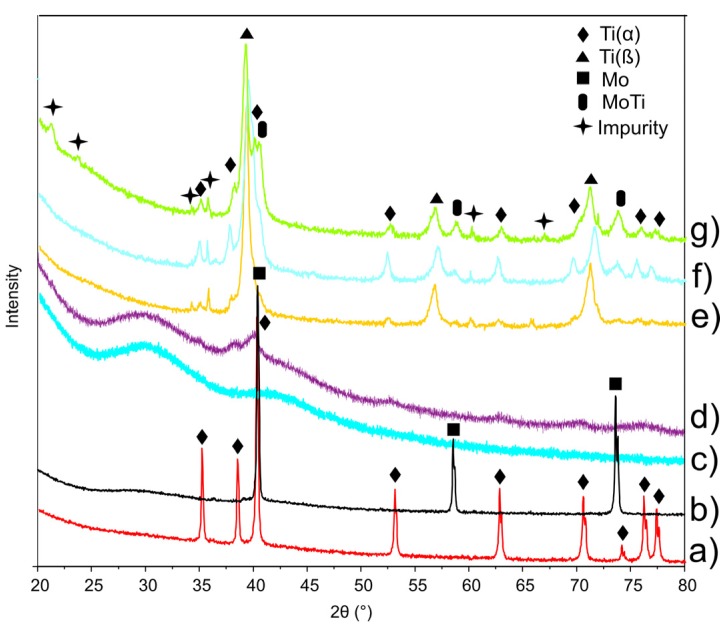
X-ray diffraction spectra of Ti (**a**); Mo (**b**) and 45S5 Bioglass (**c**) powders, their mixture after 30 h of mechanical alloying (**d**); and after annealing at 800 °C for 0.5 h: Ti23Mo (**e**); Ti23Mo 3BG (**f**); Ti23Mo 10BG (**g**).

Figure 2a,b shows the XRD patterns of the starting microcrystalline titanium (reference code 01-071-4632) and molybdenum (reference code 01-071-4645) powders. After 30 h of mechanical alloying the powders are amorphous (Figure 2d). The high plastic deformations of the powders results in high density of dislocation lines and subsequently subgrain formation, which finally leads to amorphisation [24,25]. The low temperature sintering results in crystallization and nanostructure formation.

XRD analysis of Ti23Mo (30 h MA and annealed at 800 °C for 0.5 h) showed the presence of almost single phase β-Ti type structure with cell parameter *a* = 3.234 Å (Figure 2e). When 45S5 Bioglass (Figure 2c) is added, the volume of the regular structure of Ti23Mo decreases, as manifested by a shift of the diffraction peaks of the (1,1,0), (2,0,0) and (2,1,1) crystal planes towards larger angles in comparison with pure Ti23Mo alloy (Table 1). According to the Scherrer method of XRD profiles, the mean crystallite size of heat treated Ti23Mo alloy was 8 nm. Ti23Mo 3BG nanocomposite is mostly single phase cubic β-type phase material (reference code 01-077-3482) with some impurities as α-Ti, MoTi (reference code 01-071-9821) and SiC (reference code 01-075-834; Figure 2e). With the increase of the 45S5 Bioglass contents in Ti23Mo nanocomposite increase of α-phase is noticeable.

**Figure 3:**
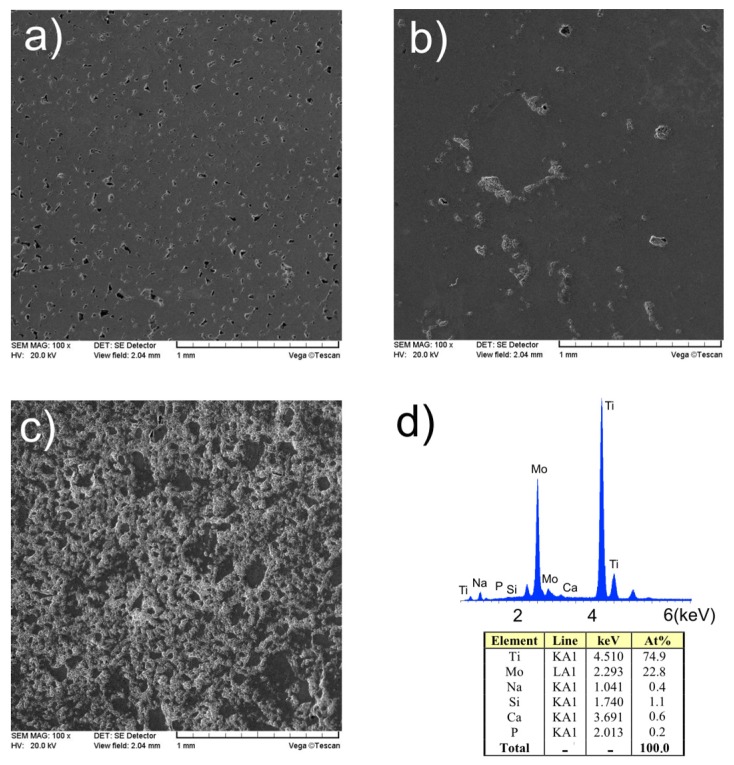
Scanning electron microscopy (SEM) micrographs of Ti23Mo alloy (**a**); Ti23Mo 3BG (**b**) and Ti23Mo 10BG nanocomposites (**c**); Energy dispersive spectroscopy (EDS) analysis of the surface of sintered Ti23Mo 3BG nanocomposite is shown in (**d**).

The smooth and porous Ti23Mo alloy surface is presented in Figure 3a. The relative density of the bulk Ti23Mo 3BG nanocomposite was measured to be 91%. The results of EDS analysis of the surface of sintered Ti23Mo 3BG nanocomposite are shown in Figure 3d. It can be confirmed that synthesized nanocomposite mainly consists of Ti-Mo matrix with elements of O, Na, Si, Ca and P.

**Table 1:** Structural parameters (*a*, *c*, *V*), crystallite size (*d*), theoretical density (ρ_th_) and microhardness (*HV*_0.3_) for bulk Ti23Mo-*x* wt % Bioglass nanocomposites.

Sample	Phase	*a* (Å)	*c* (Å)	*V* (Å^3^)	*d* (nm)	ρ_th_ (g/cm^3^)	*HV*_0.3_/10
Ti23Mo	-	3.248	-	34.265	19.1 ± 2.1	6.701	350
Ti23Mo 3BG	β	3.234	-	33.834	16.2 ± 1.5	6.581	365
Ti23Mo 10BG *	β	3.249	-	34.296	15.4 ± 0.7	-	-
	α	2.960	4.724	35.844	-	6.301	740

* 2-phase material.

**Figure 4:**
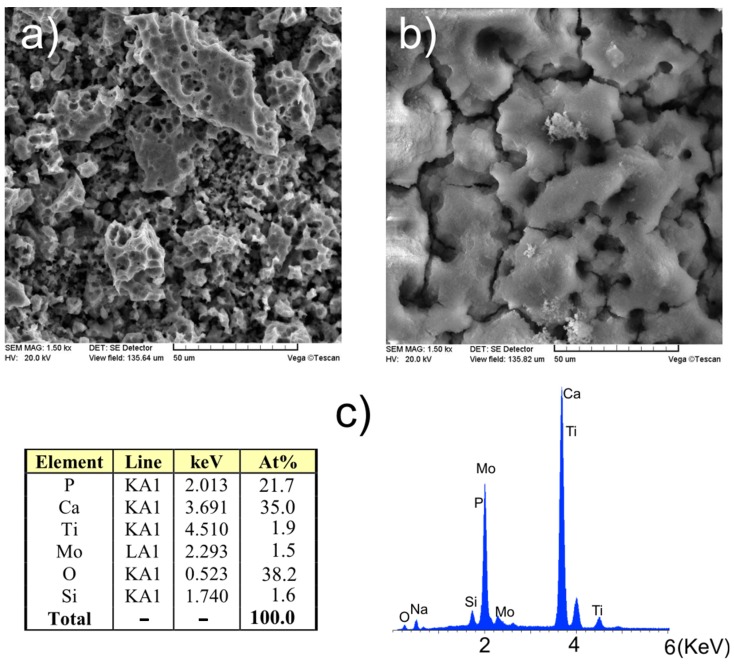
SEM micrographs of Ti23Mo 3BG after anodic oxidation (**a**) and additional Ca-P deposition (**b**) with EDS spectrum (**c**).

Electrochemical anodic oxidation results in pore formation on the surface of Ti23Mo 3BG nanocomposite. The porosity of bulk Ti23Mo 3BG nanocomposite is about 9%. In the etched surface large macropores with size up to 20 μm (Figure 4a) and smaller mesopores with size smaller than 0.5 μm are formed (not shown). The macropores are created at the places, where previously remnant pores existed as a result of the sintering, while the mesopores on the surface are the result of the large volume grain boundary etching. The prepared rough surface states a good base for tissue growth and their strong fixing or for intermediate bioceramic (Ca-P) layer formation.

Next the Ca-P deposition (Figure 4b) leads to intermediate bioactive layer formation. The electrolyte with Ca/P ratio equal to 1.67 was used. The obtained layers are porous and rough, which result in increased surface area and enlarged area for contacts with tissue. The Ca-P is deposited in pits and pores, growing into them, therefore fixating the layer in the metallic background. EDS analysis of the deposited Ca-P layer (Figure 4c) shows that Ca content is 35.00 at % and P content is 21.69 at %, which means that the Ca/P atomic ratio is equal to 1.61, which corresponds almost to the value of hydroxyapatite (reference code 00-024-0033**)**. On the spectrum, the calcium, phosphorus, and oxygen peaks dominate.

**Figure 5:**
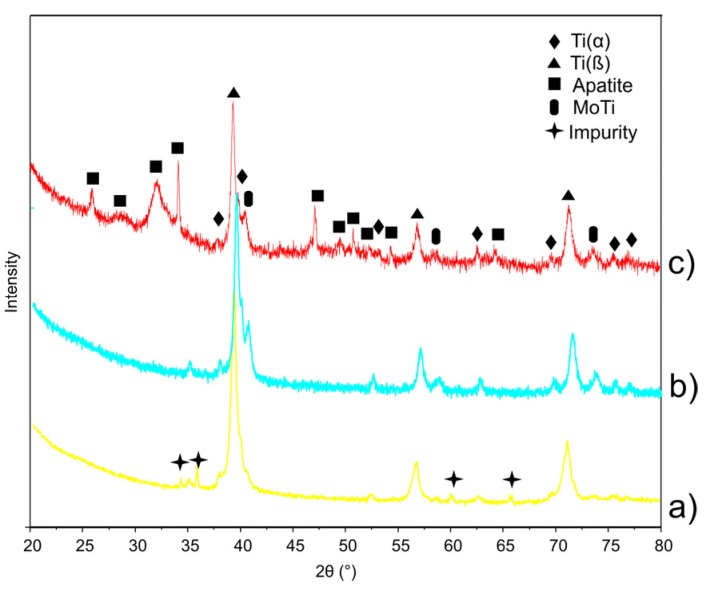
X-ray diffraction (XRD) spectra of Ti23Mo 3BG nanocomposite before (**a**) and after the surface biofunctionalization (**b**,**c**); etched (**b**) and Ca-P deposited (**c**).

XRD results (Figure 5) show sinter before Figure 5a, after electrochemical etching Figure 5b and after electrochemical etching with additional Ca-P deposition Figure 5c. The sintered Ti23Mo 3BG nanocomposite has β-type structure with some trace of α-phase. On the XRD spectrum of Ti23Mo alloy the impurity is marked (Figure 5a). For etched Ti23Mo 3BG nanocomposite SiC phase is absent, which only confirms its surface character. In the anodized surface, besides pores, α-Ti phase is formed. XRD spectrum did not reveal strong peaks belonging to titanium oxides. It is likely during the electrochemical etching process, that thin amorphous titanium oxides were formed. After Ca-P deposition, the surface layer is composed of the apatite and small trace of MoTi phase.

**Figure 6:**
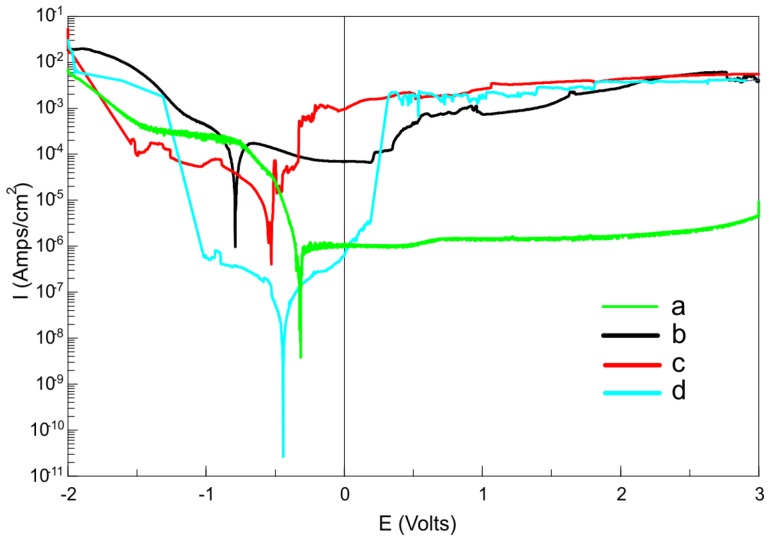
Potentiodynamic polarization curves of microcrystalline Ti (**a**); Ti23Mo 3BG nanocomposites before (**b**) and after electrochemical etching (**c**) as well as after additional Ca-P deposition (**d**) in Ringer’s solution at 37 °C.

The corrosion resistance of the different surfaces of the Ti23Mo 3BG composite in Ringer solution was investigated (Table 2, Figure 6). Using CorrView software (Solatron analytical), from the Tafel extrapolations of the recorded potentiodynamic corrosion curves, corrosion current density (*I*_cor_) and corrosion potential (*E*_cor_) were determined. The addition of 45S5 Bioglass to Ti23Mo alloy had also a positive effect on the corrosion resistance in Ringer’s solution. Ti23Mo composite with 3 wt % of 45S5 Bioglass has better corrosion resistance (*I*_cor_ = 64.44 μA/cm^2^, *E*_cor_ = −0.79 V) than nanostructured Ti23Mo alloy (*I*_cor_ = 70.98 μA/cm^2^). The best corrosion resistance is shown by Ti23Mo 3BG nanocomposite after electrochemical etching and Ca-P deposition (*I*_cor_ = 0.0168 μA/cm^2^, *E*_cor_ = −0.44 V). After the electrochemical etching the surface is much rougher in comparison to the polished one. The oxides prepared in the anodic oxidation and Ca-P phase deposition from the electrolyte on the surface of Ti23Mo 3BG nanocomposite showed improved corrosion resistance. Additionally, the corrosion potential (*E*_cor_) is shifted to the nobler direction (*E*_cor_ = −0.44 V).

**Table 2:** Corrosion current density *I*_corr_, corrosion potential *E*_corr_ and glycerol contact angles Θ of the nanocrystalline Ti23Mo and Ti23Mo 3BG nanocomposites before and after electrochemical etching as well as after additional Ca-P deposition.

Sample	*I*_corr_ (μA/cm^2^)	*E*_corr_ (V)	Glycerol Contact Angle Θ (°)
Ti23Mo	70.93	−0.79	85.64 ± 0.72
Ti23Mo 3BG	64.44	−0.79	41.41 ± 0.94
Ti23Mo 3BG etched	5.43	−0.53	26.11 ± 1.62
Ti23Mo 3BG etched and Ca-P deposited	0.02	−0.44	72.41 ± 0.56

### 3.2. Surface Properties

**Figure 7:**
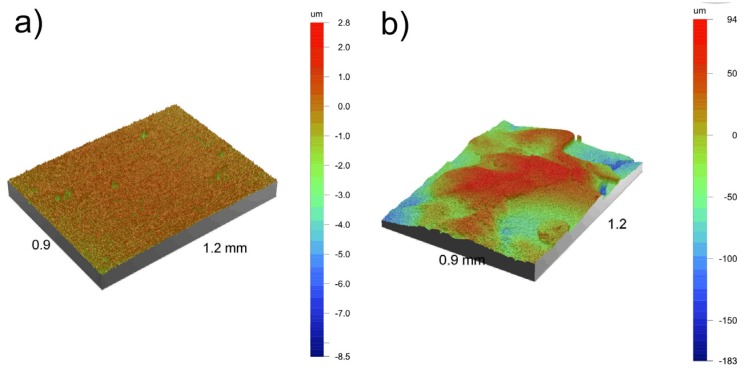
Optical profiler 3D topography of the Ti23Mo 3BG nanocomposites before (**a**) and after electrochemical etching with additional Ca-P deposition (**b**); 0.9 mm × 1.2 mm scan size.

**Figure 8:**
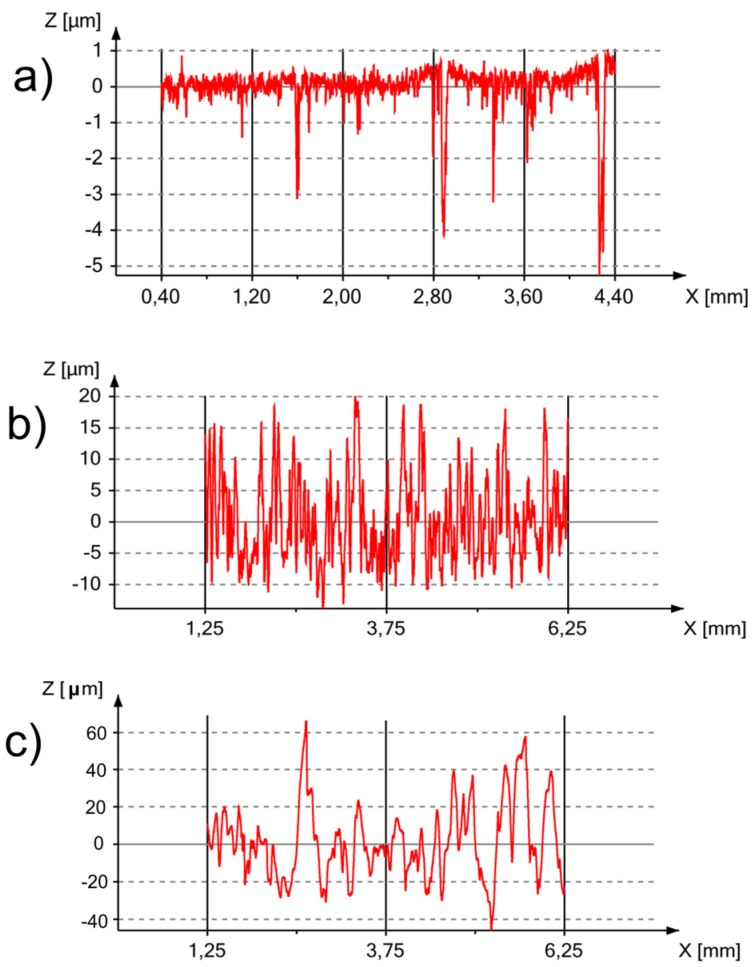
Optical profiler surface scans and X-profiles of the Ti23Mo 3BG nanocomposites before (**a**) and after electrochemical etching (**b**) as well as after additional Ca-P deposition (**c**).

Surface roughness is a considerably important property for the attachment of cells to the implant. It has been previously reported that not only micro- but also nano-topography can support the proliferation of different types of cells [12]. Optical profiler 3D topography and profiler surface scans and X-profiles of the bulk Ti23Mo 3BG nanocomposite, at the different processing stages, are presented in Figure 7 and Figure 8, respectively.

At different processing stages changes occur in the surface porosity (Figure 7). Primarily the polished surface exhibited only few pores (Figure 7a), which resemble the remnants of the sintering process. Significant differences were observed after etching and additional Ca-P deposition (Figure 7b). The increase of surface together with surface composition modification plays a key role for the living cell attachment and proliferation.

The bulk Ti23Mo 3BG nanocomposite had *R*_a_, *R*_t_ and *R*_z_ values of approximately 0.29, 11.29 and 8.24 μm, respectively (Table 3). In contrast, all of the roughness parameters increased for the etched and Ca-P deposited Ti23Mo 3BG nanocomposite surface (Figure 7b). This sample surface had an average surface roughness with *R*_a_, *R*_t_ and *R*_z_ values in the range of 35.80–276.55 μm. The optimal pore size for the cell attachment, differentiation, and ingrowth of osteoblasts and vascularization has been reported to be approximately 200–500 μm [26].

In Figure 8, the respective X-line profiles of the studied materials are shown. Large pores are formed in the surface during the etching process. This surface is able to support cell growth and proliferation, which provides a surface with large hillocks and a very non-uniform surface profile after culturing of the osteoblasts. The profile is an effect of the multilayered nature of growing cells.

**Table 3:** 2D (*R*_a_, *R*_t_, *R*_z_) and hybrid (*S*_dq_, *S*_dr_) parameters for the Ti23Mo 3BG nanocomposite on different processing routes; parameters taken from surface area of 1.08 mm^2^.

Processing Route	*R*_a_ (μm)	*R*_t_ (μm)	*R*_z_ (μm)	*S*_dq_ (°)	*S*_dr_ (%)
before etching *	0.29	11.29	8.24	8.97	1.22
after etching	6.42	42.47	35.54	74.37	341.72
after etching with Ca-P deposition	35.80	276.55	247.74	69.84	255.66

* polished.

The hybrid parameters (*S*_dq_, *S*_dr_,) reflect slope gradients and exhibit comparable behavior to the roughness parameters. The *S*_dq_ value that affects the wetting of the surface by fluids significantly increases for the etched Ti23Mo 3BG sample. An increase in the surface slope (*S*_dq_) may improve the fixing force of the cells, which ultimately provides a more stable connection between the implant and bone. Finally, *S*_dr_, which expresses the increase of the interfacial surface area relative to the area of the projected (flat) *x*, *y* plane, may improve the area for the attachment of cells. In our case, there is a considerable increase of *S*_dr_ for the etched and Ca-P deposited Ti23Mo 3BG surface (255.66%) compared to the polished surface (1.22%).

**Figure 9:**
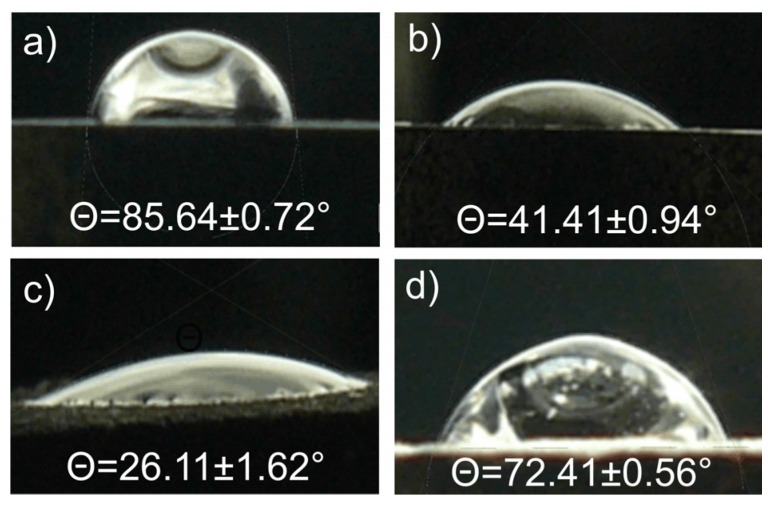
Contact angles of glycerol on the nanostructured bulk Ti23Mo alloy (**a**) and Ti23Mo 3BG nanocomposite before (**b**) and after electrochemical etching (1 M H_3_PO_4_ + 2% HF; 10 V/30 min) (**c**) as well as after additional Ca-P deposition (**d**).

As shown in Figure 9 surface wettability assay recorded a lower glycerol contact angle of Ti23Mo 3BG bulk nanocomposite (41.41° ± 0.94°) than that of the nanocrystalline Ti23Mo alloy (85.64° ± 0.72°). Etched nanocomposite exhibited the enhanced surface hydrophilicity (26.11° ± 1.62°). Additional Ca-P deposition after electrochemical etching showed, that contact angle significantly increases (72.41° ± 0.56°), which means that wettability decreases (Table 2). The change of two or more surface characteristics at the same time, such as surface roughness and chemistry, whether deliberate or not, complicates the evaluation of the roles of the parameters on the wetting behavior and the biological performance [27].

### 3.3. In vitro Cytocompatibility

**Figure 10:**
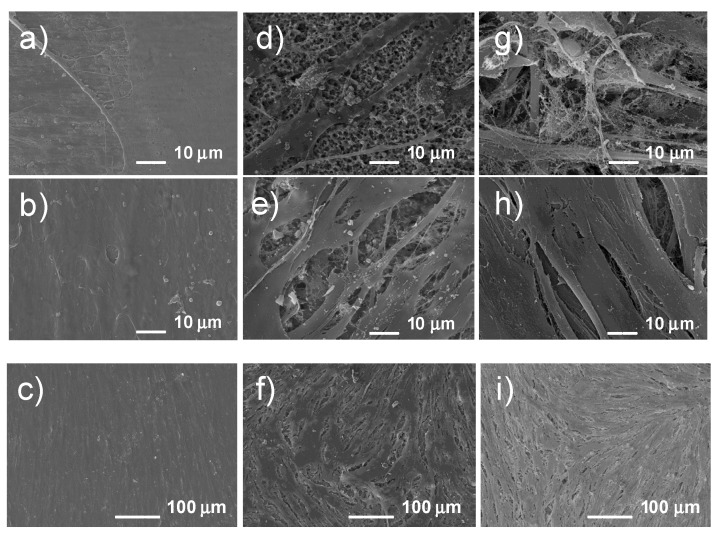
Osteoblast culture on the surface of the Ti23Mo 3BG nanocomposite before etching (**a**,**b**,**c**), after etching (**d**,**e**,**f**) and after etching with additional Ca-P deposition (**g**,**h**,**i**) after 1st (**a**,**d**,**g**) and 5th day ((**b**,**c**), (**e**,**f**) and (**h**,**i**)—different magnifications).

The culture was prepared on the Ti23Mo 3BG nanocomposite sample before electrochemical etching (Figure 10a–c), after electrochemical etching only (Figure 10d–f) and after etching with Ca-P deposition (Figure 10g–i). The collected data reveals a significant difference in the morphological characteristics of the cells on the porous and polished materials even after the first day of cell culturing (Figure 10). The surface irregularities (protrusions, hillocks and pores) improve osteoblast adhesion, thus cells attach on the micro- and nano-surface irregularities. It has been proven that cell attachment and proliferation depend on the surface topography and roughness [28]. The osteoblasts that grew on the Ti23Mo 3BG nanocomposite sample before electrochemical etching exhibited adhesion to the material surface after one day and covered most of the surface after five days (Figure 10a–c). In the case of the electrochemically etched sample, after 24 h of incubation, cells penetrated into the structure of the surface instead of forming filapodia (Figure 10d). After culturing the cells for five days, the cells on the etched Ti23Mo 3BG nanocomposite were observed to be well spread, both on the surface of the porous scaffold and inside the pores (Figure 10e,f).

On the other hand, in the case of the etched and Ca-P deposited Ti23Mo 3BG sample, after the first day of incubation, cells showed good adhesion to the surface of the studied sample in the form of filapodia (Figure 10g). A monolayer was formed on the surface of the sample after five days of incubation (Figure 10h,i). The covering of the implant surface with Ca-P layer is the final stage of the surface treatment.

## 4. Discussion

Nanotechnology is used for improving biomaterials or creating new biomaterials used for specific dental applications [29]. Nanostructured biomaterials can be personalized by engineering their structure, shape, size, and surface properties in order to be applied in precise anatomical sites. Based on the *in vitro* results, nanostructured metals undoubtedly have the potential to yield a faster and more stable integration of dental implants [29,30,31]. The *in vivo* effects of several nanoscale surface modification approaches and a potential applicability of these techniques to already commercialized dental implants are currently under investigation [28,29].

Current research focuses on improving the mechanical performance and biocompatibility of metal-based systems through changes in alloy composition, microstructure, and surface treatments [12,15,22,26,32,33]. In the case of titanium, a lot of attention is being paid to enhance the strength characteristics of commercial purity grades in order to avoid potential biotoxicity of alloying elements, especially in dental implants [34,35].

Improvement of the physicochemical and mechanical performance of Ti-based implant materials can be achieved through microstructure control, the top-down approach known as mechanical alloying technique [13,14,15,16,17,22,36,37]. Recent studies showed clearly that nanostructuring of titanium can considerably improve not only the mechanical properties, but also the biocompatibility [33]. Nanostructured materials can exhibit enhanced mechanical, biological, and chemical properties compared with their conventional counterparts [30,31,37].

While these biomaterials have been successful in encouraging bone ingrowth both *in vivo* and in clinical trials, the range of materials and microstructures available is still rather limited. To optimize Ti-based alloys for dental implant applications, several studies have focused on the design of biomaterials with optimized architecture to fulfill physico-chemical, mechanical as well as regeneration requirements. β-type titanium alloys are a class of interesting biomaterials that can exhibit a unique combination of physical, chemical and mechanical properties [38].

In this work, mechanical alloying was used to synthesize nanostructured Ti23Mo xBG nanocomposites. This technique enables alloying of elements that are difficult or impossible to combine by conventional melting methods (*i.e.*, Ti and Bioglass). The nanocomposite surface improvement was achieved by electrochemical treatment. This process was composed of two stages: anodic oxidation and Ca-P deposition. The applied etching electrolyte was composed of a mixture of 1M H_3_PO_4_ and 2% HF. Four stages of anodic oxidation were suggested [39]: (i) a compact oxide barrier layer formation; (ii) field-enhanced dissolution of the oxide layer; (iii) formation of porous structure in the slits and cracks (pore formation and dissolution of the oxide layer is possible as well) and (iv) the porous structure is consumed when the dissolution rate is larger than the pore formation rate.

It has been proven that the surface properties of the Ti-based implants determine successful osseointegration. The results published recently by Sul *et al.* showed that the morphology of different commercially available clinical titanium implants differed due to the surface modification techniques used during manufacture [40]. The bulk nanostructured Ti23Mo 3BG nanocomposite had *R*_a_, *R*_t_ and *R*_z_ values of around 0.29, 11.29 and 8.24 μm, respectively. The etched Ti23Mo 3BG composite had average surface roughnesses, with *R*_a_, *R*_t_ and *R*_z_ in the 6.42–42.47 μm range. After the Ca-P layer deposition a still relatively rough surface with Ca-P protrusions is well visible.

Implants, due to the corrosive environment of the tissue and body fluids, may undergo unexpected local corrosion attacks, leading to a release of the corrosion products to the tissue and its poisoning. The corrosion test results indicated that the nanostructured Ti23Mo alloy possesses a lower corrosion resistance and consequently a higher corrosion current density (*I*_corr_ = 70.93 μA/cm^2^, *E*_corr_ = −0.7925) in the Ringer’s solutions. The bulk Ti23Mo 3BG nanocomposite has a better corrosion resistance (*I*_corr_ = 0.0168 μA/cm^2^, *E*_corr_ = −0.4423). For comparison, corrosion current density of microcrystalline Ti is 0.658 μA/cm^2^ in Ringer’s solution at 37 °C.

The surface wettability was confirmed to be a key parameter influencing the proliferation of osteoblasts [27,41]. Enhanced surface wettability would improve the cellular adhesion by optimizing the protein adsorption and providing a suitable surface presented to the filopodia. As a result, the electrochemically etched Ti23Mo 3BG nanocomposite, due to its good corrosion resistance and the surface wettability (contact angle Θ = 26.1°), shows a high extent of cell proliferation.

In order to test the toxicity of materials, as well as interaction between the tested materials and the cells, *in vitro* cytocompatibility tests are conducted. The first results of increased osteoblast adhesion on the nanostructured metals was published by Webster and co-workers [12,26,30,31]. In this work, the *in vitro* cytocompatibility test was performed on the samples with different surfaces. The residual porosity after powder compaction played an important role in the cell adhesion. A significant difference in the morphological characteristics of the cells on the etched and polished materials was visible after one day of cell culturing. The cells tend to adhere with their entire surface to the porous sample penetrating the pores, whereas more spherical cells were seen with a smaller contact surface but more filapodia on the polished surface. After the first day, the cells strongly attached to the surface of the etched nanocomposite Ti23Mo 3BG sample, not producing filapodia or only producing them in a smaller amount compared to the cell culture on the smooth surface. After five days of cell culture, a smaller portion of the observed area was covered by cells on the etched Ti23Mo 3BG composite in comparison with the analogous bulk sample.

Bioactive glasses are biocompatible and exhibit a strong interfacial bonding with bone. Their bioactivity is attributed to the formation on their surface of a hydroxycarbonated apatite (HCA) layer. The rate of tissue bonding appears to depend on the rate of HCA formation, which follows a sequence of reactions between the implanted material and the surrounding tissues and physiologic fluids [42,43,44,45]. Cao and Hench proposed a three-step mechanism of HCA formation when the bioactive glass comes into contact with physiologic fluids: ion exchange, dissolution, and precipitation [42]. In the last case, precipitation of the calcium and phosphate ions released from the glass together with those from the solution, form a calcium-phosphate-rich layer (CaP) on the surface [44].

Modification at the nanoscale of the surface properties of dental implants can be achieved by various techniques, in order to create a more efficient implant integration with the bone [46]. Alterations in the cell shape and cytoskeleton, thus influencing specific gene expression, have been noticed after modifying the surface texture or roughness of the implants [46,47]. Moreover it has been demonstrated that anodized titanium surfaces enhances osteogenic activity *in vitro* [48,49]. On the other hand, oxidative nanopatterning confers titanium-based metals the ability to selectively guide cell behavior, facilitating the growth of cells having the osteoblastic potential [50]. Therefore these techniques are crucial for tissue regeneration, leading in the future to higher predictability in tissue healing around dental implants.

## 5. Conclusions

In this work, new kinds of biomedical Ti23Mo xBG nanocomposite were developed by the introduction of 45S5 Bioglass powders into the Ti23Mo matrix. Additionally the formation of a porous nanocrystalline Ti23Mo 3BG nanocomposite with an electrochemically modified surface was demonstrated. On the etched surface a bioactive HA layer was deposited. The following conclusions can be withdrawn: (1) etching improves porosity of the surface; (2) Ti23Mo 3BG nanocomposite after etching and Ca-P deposition had an average surface roughness in the 35.80–276.55 μm range; (3) the corrosion resistance significantly changes after the electrochemical treatment and Ca-P deposition; (4) noticeable difference in the morphology of the NHOst cells on porous and polished materials is observed in SEM, even after one day of cell culturing; (5) the rough, electrochemically biofunctionalized surface (porous with Ca-P layer) supports osteoblast cell growth and proliferation.

The key factors for the success of implant integration with the surrounding hard tissues are surface topography and chemical composition. Therefore the biofunctionalized nanocrystalline Ti23Mo 3BG composite may be an important step forward in the development of such a structure, which will support the process of osseointegration of the implant.
